# Do Payments Pay Off? Evidence from Participation in Costa Rica’s PES Program

**DOI:** 10.1371/journal.pone.0131544

**Published:** 2015-07-10

**Authors:** R. A. Arriagada, E. O. Sills, P. J. Ferraro, S. K. Pattanayak

**Affiliations:** 1 Millennium Nucleus Center for Socioeconomic Impact of Environmental Policies (CESIEP), Center of Applied Ecology and Sustainability (CAPES), Department of Agricultural Economics, Pontificia Universidad Católica de Chile, Avenida Vicuña Mackenna 4860, Macul, Santiago, Chile; 2 Department of Forestry and Environmental Resources, North Carolina State University, Raleigh, North Carolina, United States of America; 3 Department of Economics, Andrew Young School of Policy Studies, Georgia State University, Atlanta, Georgia, United States of America; 4 Sanford School of Public Policy, Duke University, Durham, North Carolina, United States of America; 5 Nicholas School of the Environment, Duke University, Durham, North Carolina, United States of America; University of New South Wales, AUSTRALIA

## Abstract

Payments for environmental services (PES) are often viewed as a way to simultaneously improve conservation outcomes and the wellbeing of rural households who receive the payments. However, evidence for such win-win outcomes has been elusive. We add to the growing literature on conservation program impacts by using primary household survey data to evaluate the socioeconomic impacts of participation in Costa Rica’s PES program. Despite the substantial cash transfers to voluntary participants in this program, we do not detect any evidence of impacts on their wealth or self-reported well-being using a quasi-experimental design. These results are consistent with the common claim that voluntary PES do not harm participants, but they beg the question of why landowners participate if they do not benefit. Landowners in our sample voluntarily renewed their contracts after five years in the program and thus are unlikely to have underestimated their costs of participation. They apparently did not invest additional income from the program in farm inputs such as cattle or hired labor, since both decreased as a result of participation. Nor do we find evidence that participation encouraged moves off-farm. Instead, semi-structured interviews suggest that participants joined the program to secure their property rights and contribute to the public good of forest conservation. Thus, in order to understand the social impacts of PES, we need to look beyond simple economic rationales and material outcomes.

## Introduction

Over the last two decades, payments for ecosystem services (PES) have gained prominence as a strategy to protect ecosystem services and mitigate climate change through reduced emissions from deforestation and forest degradation (REDD) [1]. PES programs typically make financial transfers to landholders conditional on their adoption of land use practices believed to generate ecosystem services [2–4], and they are recommended when the public net benefits of those practices are much larger than the private net costs [5]. Because the financial transfers enable landholders to capture some of the external value of the ecosystem services that they produce [6], PES have also been viewed as a tool to increase the welfare of participating landholders, or at least ‘do no harm’ while inducing conservation, as called for under REDD safeguards [3,7–10]. As a result, many developing countries have incorporated PES programs into their “portfolio of rural development programs and projects” [11].

The impacts of PES on rural development depend on both who receives payments and how recipients are affected by participation in the program. There is a large literature describing who participates in PES programs (e.g. for Costa Rica, see [12–14]), including guidance on how to reduce barriers to participation by the poor [15]. There have been fewer studies of how recipients are affected by participation in PES, perhaps because it seems obvious that recipients would not voluntarily participate if they did not benefit from the program.

When participation in PES is truly voluntary, one can plausibly assume that participants believe they benefit from participation (particularly when they renew their contracts). Nevertheless, clarifying how exactly participants benefit and how their livelihoods are affected by participation are important empirical issues, for both welfare and conservation outcomes [16,17]. Recent reviews by [1] and [18] conclude that there is insufficient evidence about the conditions under which PES has positive socioeconomic impacts.

To help build the evidence base, we examine how landowners were affected by participation in Costa Rica’s renowned Program of Payments for Environmental Services (*Programa de Pagos por Servicios Ambientales*, or PSA) in the first decade of its implementation. Using household data and a quasi-experimental design, we find no impact of PSA on household wellbeing, as measured by an asset index and self-reported wellbeing. We therefore consider other possible uses of the cash income that landowners receive from PSA, including investment in farm inputs or financing a move off-farm. However, we find that participation had negative effects on two key farm inputs in this region (size of the cattle herd and probability of hiring farm labor) and no effect on the probability of living off-farm. This leads us to consider other possible reasons why landowners enrolled their forestland in PSA by revisiting lessons from case studies conducted in our study site using mixed methods to understand program participation [19].

## Materials and Methods

### PES as implemented in Costa Rica

The Costa Rican government is well-known for its conservation policies, including its large protected area network, support for private reserves, efforts to control illegal logging, and PSA [20]. Initiated in 1997, PSA has been financed by an earmarked gasoline tax, international donors and ecosystem service buyers [21]. Since 1997, PSA has offered several different types of contracts to landowners, including some that support reforestation and forest management, continuing previous policies of financial incentives for forest management [22]. However, PSA is best known for offering direct payments to private landowners for forest conservation, and we estimate the impacts of signing one of those contracts.

Forest conservation contracts are intended to remunerate landowners for the ecosystem services provided by their forests, including climate change mitigation, watershed functions, biodiversity and scenic beauty [23]. Although landowners are paid for the area of forest under contract, rather than for service flows, the law establishing PSA was framed explicitly in terms of compensation for the provision of ecosystem services. Lawmakers believed this framing made the importance of forest conservation more obvious and relevant to stakeholders cf. [24]. Another key motivation for establishing PSA was to increase rural income, especially of small and medium size landowners [23,25].

Implementation of PSA has varied over time and across regions [8,15,21,22,19,26]. Prior to 2000, application to the program required an official cadastral map from the National Land Registry, a cartographic map indicating the location of the forest parcel proposed for PSA payment, a forest management plan, and proof of land ownership [27]. Applications were accepted on a “first come, first served” basis. Upon acceptance, a contract was signed for a designated forest parcel, obligating the landowner to preserve that area by fencing and posting signs, preventing forest fires and hunting, and refraining from any agricultural use or wood extraction. In return, the government makes annual payments per hectare for five years, with the potential for renewal. The government also bears significant transactions costs, although it has sought to limit those by offering a uniform annual payment per hectare on all forest conservation contracts [28].

There is an extensive and dynamic literature about PSA, largely focused on its conservation impacts but also considering how landholders are selected and affected by participation. Most evaluations have found that PSA promotes forest conservation, although the increase in forest cover that can be attributed to the program is much less than the area under contract [4,10,27]. Legrand et al. [23] concluded that PSA has positive environmental impacts, but has tended to exclude small landowners. In contrast, [29] determined that 58% of all PSA forest conservation contracts issued between 2001 and 2005 were directed to small and medium landowners. Milder et al. [30] argued that PSA provides important livelihood benefits to participating landowners through both cash payments and noncash benefits such as enabling the transition to more profitable and resilient land-use systems, securing land tenure, and strengthening social capital and supportive local institutions. Consistent with this, [31] concluded that PSA participation accelerated farmers’ exit from agriculture in the Osa Peninsula. Several authors have claimed that participation in PSA can improve tenure security by documenting that land kept under forest is being used to produce ecosystem services, rather than being ‘idle’ and therefore legally vulnerable to squatters [13,19].

Some authors have tried to quantify the importance of the payments within the household budgets of program beneficiaries. Based on a survey of households in the Central Volcanic Cordillera region, [13] estimate that payments comprise about 16% of household budgets, but they also note that the percentage is smaller (5%) for landowners with small areas under PSA contracts. Based on another survey, [12] estimates that payments make up about 10% of household budgets. In contrast, [32] reports much higher percentages in the Osa region, where landowners are poorer. Most of these studies draw their conclusions from small samples of landowners who are not necessarily representative of PSA participants in a given region, e.g. oversampling land owners who derive most of their income from off-farm sources.

In sum, the literature suggests a number of ways that participation in PSA can affect landowners, but there have been few attempts to quantify the causal impacts. In particular, no previous studies have employed a rigorous quasi-experimental design to address the fundamental evaluation question of what would have happened to participants if they had not signed a PSA contract?

### Conceptual Framework

**Fig 1:**
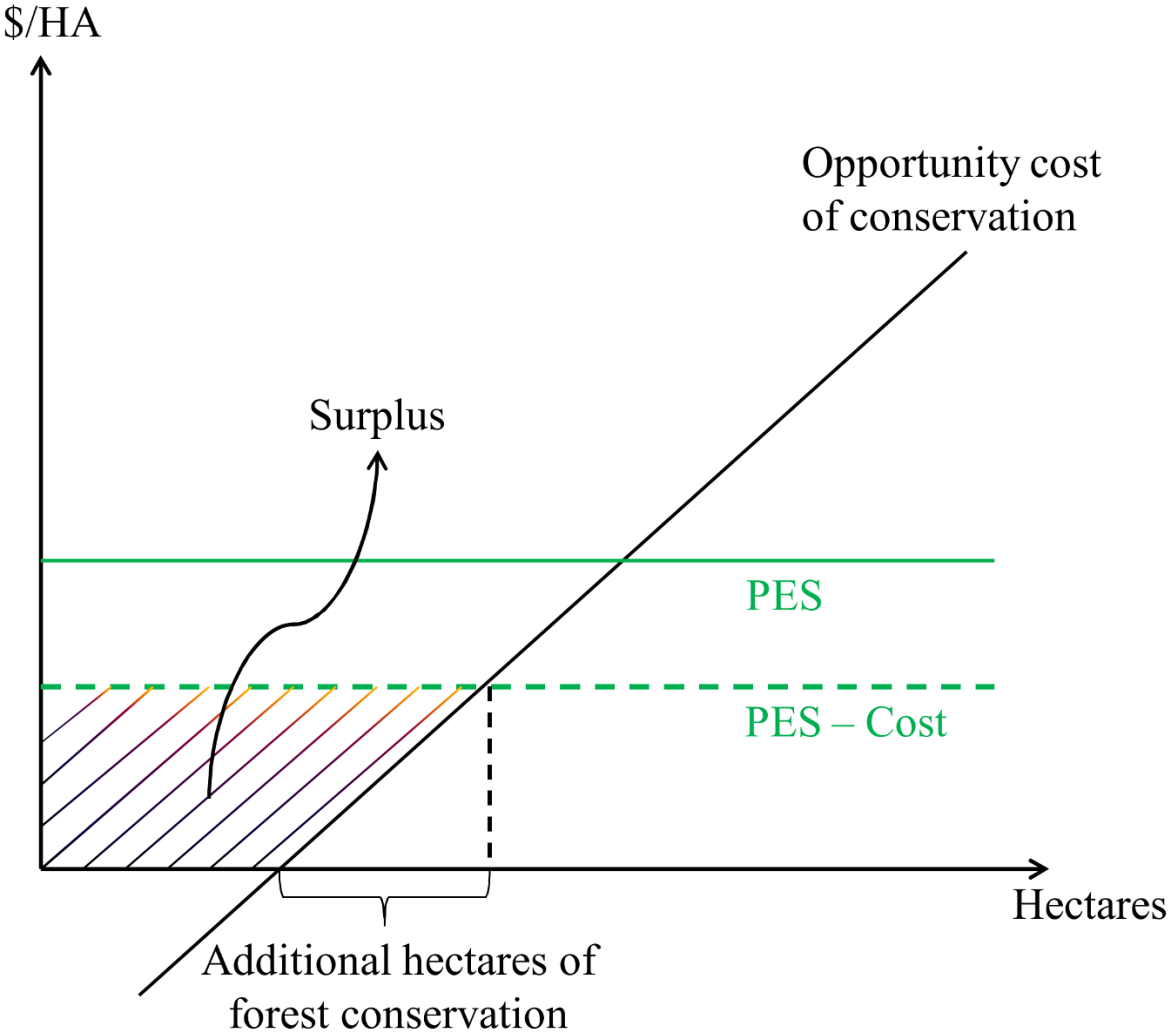
Landowners responses to PES.

In most landscapes undergoing deforestation, only a fraction of the forests will be cleared at any given time, reflecting the underlying heterogeneity in returns from deforestation. Fig 1 represents this heterogeneity by arranging all forest lands from least to highest returns to deforestation, or equivalently opportunity costs of conservation. Forested lands with the lowest returns are the least likely to be deforested and most likely to be placed under contract in a voluntary PES program with uniform payments [2,10,33–36]. Some payments thus inevitably go to contracts to conserve forests that would have been conserved without payments. Evidence for this phenomenon can be found in our study sample. Sample farms with PSA contracts are an average of 165 ha in total, with 76 ha under contract. Arriagada et al. [27] estimate that these contracts induced, on average, about 10 ha of additional forest cover during the first eight years of the program. PSA was clearly making payments on forested hectares that would not have been converted without payments. This implies that participants earn windfall gains, shown as the area of surplus in Fig 1.

These potential windfall gains can be reduced by contractual requirements of the program (e.g. developing a certified forest management plan, posting and clearing the boundaries of the forested parcel), which effectively reduce the net benefit per hectare of participation. However, with the additional (plausible) assumption that the supply curve (marginal cost curve) of land for PSA contracts is upward sloping, participants should earn a surplus from *most* hectares enrolled in any voluntary, uniform-payment PES program [34]. These “excess payments” may be desirable as part of a rural development strategy, or as a political strategy to reduce opposition to land-use restrictions. Regardless, they represent a real, risk-free transfer to landowners who participate in the program. Participants may use these transfers for a variety of purposes, including to increase current consumption or to invest in durable consumer goods.

The likelihood of such transfers explains why PES programs are widely expected to improve the wellbeing of program participants. PES makes most sense as a policy for *both* conservation *and* rural development if it induces investment in household assets, such as durable consumer goods, that will increase well-being over the long-run. However, for any given household and any given parcel, PES may or may not be enough to compensate for lost income and contract compliance costs. If potential participants have imperfect information or face external pressures to participate, the result could be an increase, decrease, or no net change in household income. Further, any increase in income could be allocated differently by different households, with implications for their well-being and land use. For example, participants could use payments to finance (i) off-farm activities that reduce deforestation pressure cf. [17,31] or (ii) purchase of farm inputs such as livestock and hired labor, consistent with slippage of deforestation to land not under contract cf. [37]. In addition to the implications for forest conservation, using payments for these types of investments may mean no detectable short-to-medium run impacts on household well-being.

### Sampling and data collection

Using household-level data collected in 2005 (S1 File), we evaluated the impacts of PSA contracts in three (out of 4) cantons in the region of *Sarapiquí* located in northeastern Costa Rica: *Sarapiquí*, *Guacimo*, and *Pococí*. These are among the poorest cantons in Costa Rica, ranked 65^th^ (*Guacimo*), 68^th^ (*Sarapiquí*), and 69^th^ (*Pococí*) out of 81 cantons in Costa Rica [38]. Focusing on a particular region made it feasible to directly survey landowners rather than rely on secondary data. *Sarapiquí* is an ideal region for the study not only because of its economic development needs, but also because of the presence of the intermediary organization FUNDECOR. FUNDECOR has been actively involved in the design of the conservation and development strategy for the entire *Cordillera Volcánica Central* region [39], and maintains good records on participants, including farm locations.

#### PSA outcomes

Our unit of analysis is the household. Table 1 lists the outcomes that we consider, starting with changes in a standard asset index cf. [40,41]. Of course, participants could use the cash income from PSA to meet immediate consumption needs, rather than investing in assets. We expect both of these to be reflected in perceived changes in well-being. Alternatively, payments could be used to finance a move off-farm or to purchase farm inputs, so we also estimate impacts on the probability of living off-farm and on two key farm inputs in this region: cattle and hired labor. We note that these different potential uses of the payments have different implications for land use. For example, the conservation goals of the program could be furthered by households moving off-farm cf. [31] or undermined by expansion of the cattle herd.

**Table 1:** Description and summary statistics for outcomes (imputed dataset with N = 202 including 50 PSA farms). Note: Groups are different at the 95% significance level whenever the t-stat is greater than 1.96 or the normalized difference is greater than 0.400 (Imbens and Wooldridge, 2009).

Category	Description	Mean PSA(*n* = 50)	Mean non-PSA(*n* = 152)	t-stat	Norm Diff^b^
Changes in quality of life	Change in asset index (2005 Index—1996 Index)^*a*^	0.97	1.18	1.18	0.20
	Change in asset count (2005 count—1996 count)	1.66	2.03	1.18	-0.20
	Stated welfare change since 1996 (dummy variable: 1 indicates better quality of life in 2005 compared to 1996)	0.88	0.94	1.51	-0.22
Changes in livelihoods^c^	Change in absentee status since 1996 (dummy variable: 1 indicates living on-farm in 1996 and living off-farm in 2005)	0.09	0.08	-0.21	0.03
	Change in cattle owned between 1996 and 2005	-0.69	13.91	2.45	-0.44
	Change in hired labor since 1996 (dummy variable: 1 indicates no hired labor in 1996 and hired labor in 2005)	0.06	0.28	3.50	-0.64

^*a*^ This index of socioeconomic status is the first principal component of indicators for ownership of different asset classes (car, motorcycle, bicycle, landline phone, mobile phone, television, microwave, refrigerator or radio).

^***b***^ Normalized difference = X¯T− X¯CST2+ SC22. where T = PSA and C = non-PSA [64].

^*c*^ These changes also represent possible mechanisms for the impact of PSA on forest cover (Arriagada et al. 2012).

Changes in wellbeing: Asset ownership is frequently used to assess the welfare status of rural households in developing countries [42]. In particular, when income and expenditure data are not available, household ownership of consumer durables may be employed to describe socio-economic wellbeing. Ownership of each asset can be examined separately or used to construct indices with either equal or variable weights on ownership of the component assets [43]. In addition to measuring asset ownership, we also directly ask households about their subjective perceptions of changes in their quality of life. The specific variables are as follows:

- Changes in an asset index: the difference in household assets owned in 2005 and 1996 (in Spanish: *de la siguiente lista que le voy a mencionar*, *¿cuáles de ellas tiene usted actualmente que todavía funcionan*? *¿Y cuales tenía en 1996*?). Based on the responses, an asset index was calculated as the first principal component of indicators for ownership of different asset classes (i.e. car, motorcycle, bicycle, landline phone, mobile phone, television, microwave, refrigerator or radio).
- Changes in asset count: the difference in the sum (count) of the categories of assets owned by the family in 2005 and 1996.
- Changes in quality of life: self-reported change in the quality of life between 2005 and 1996 (better, same, or worse; in Spanish: *pensando en la calidad de vida global de su familia*, *comparado a 1996*, *¿usted cree que está mejor*, *igual o peor ahora*?).

Changes in livelihoods: Sierra and Russman [31] suggested that one pathway for PSA to impact forest cover is by enabling farmers to move off their farms. On the other hand, there have been concerns raised that PES could generate leakage by allowing farmers to intensify production on farm parcels not under contract [44]. In *Sarapiqui*, farmers could intensify production by investing in either cattle ranching or crops. Thus we measure:

- Residence: the difference in residence (whether live off-farm) in 2005 and 1996 (in Spanish: *¿usted vive en la finca actualmente*?*¿usted vivía en la finca en el año 1996*?).
- Changes in cattle owned: the difference between the numbers of cattle owned in 2005 and 1996.
- Changes in hired labor: the difference in whether hired non-family labor in 2005 and 1996.

#### Treatment and control groups

The sample of PSA participants comprises farmers who signed 5-year contracts for forest conservation during the first two years of the PSA and subsequently renewed their contracts. This sample gives us the longest possible period to assess impacts based on a survey conducted in 2005. Contracts could be established on properties of up to 300 ha. FUNDECOR served as an intermediary for 70 forest conservation contracts that were signed in 1997 or 1998 and were still in force in 2005. From that population of contracts, 50 were randomly selected. On average, each participating household received a PSA cash payment of over $3000 per year (forest conservation payment of $43/ha times average of 76 ha of forest land under PSA contract).

We obtained a similar sample of 150 farms without PSA contracts, in order to form a comparison group. To reduce the potential for selection bias in our estimators of impacts, we selected nearby farms using three methods: (1) geographic rule for interviewers to identify immediate neighbors of participants; (2) random sample stratified by district of participants, using the National Land Registry as the sampling frame; and (3) random sample stratified by buffer rings around each PSA property, using the National Land Registry to establish a sampling frame of all farms located in a buffer ring with inner radius of 1,920 meters and outer radius of 3,840 meters [27,45].

Landowners in the comparison group were screened out if they had since signed a PSA forest conservation contract or if they had been ineligible to receive a PSA forest conservation contract in 1997 (e.g., due to property size, tenure status, or lack of forest). Specifically, we excluded parcels in the National Registry that were smaller than 5 ha (the minimum contract size among our sample of participants for those years), parcels listed in FONAFIFO’s records as having PSA conservation contracts sometime between 1997 and 2005, and parcels owned by the state and large companies ineligible for PSA. For the district and buffer samples, three landowners were selected at random for each participant. If the interviewer failed to find the first landowner after three documented attempts or if the landowner was ineligible for a PSA contract, the next landowner on the list was sought until an interview was obtained (except for a few cases in the district stratified sample in which none of the three landowners could be located). Our final control group comprised 51 immediate neighbors, 58 landowners with properties located in buffers around each PSA property, and 43 landowners in the sample stratified by districts. Table 1 and the “unmatched” rows of Table 2 present summary statistics for the variables used in this analysis.

**Table 2:** Covariate Balance. Note: The seventh and eighth columns present three measures of the differences in the covariate distributions between PSA and non-PSA farms. If matching is effective, all of these measures should move dramatically toward zero (Ho et al., 2007).

Variable ^*a*^	Sample ^*d*^	Mean Value PSA	Mean Value Non-PSA ^*e*^	Diff Mean Value	p-value	Raw eQQ Diff ^*f*^	Mean eCDF Diff ^*g*^
Total native forest in 1992 (ha)	UnmatchedMatched	86.1351.73	37.14 45.89	48.99 5.84	0.02 0.50	45.82 8.79	0.14 0.05
Farm size (ha)	UnmatchedMatched	165.1181.08	71.43 73.78	93.68 7.31	0.06 0.64	90.05 18.39	0.19 0.10
D—Previous participation in other forest programs	UnmatchedMatched	0.32 0.25	0.25 0.25	0.07 0.00	0.01 1.00	0.18 0.00	0.09 0.00
Distance to forestry office (km)	UnmatchedMatched	29.48 30.64	25.08 28.21	4.41 2.43	0.05 0.19	4.68 3.82	0.08 0.05
Percent farm on steep slope ^*b*^	UnmatchedMatched	38.40 37.25	25.54 34.01	12.86 3.24	0.01 0.28	13.01 4.49	0.13 0.05
D—Forest fenced in 1996	UnmatchedMatched	0.20 0.15	0.50 0.15	-0.30 0.00	0.00 1.00	0.30 0.00	0.15 0.00
Hectares of forest in 1992 minus hectares of forest in 1986	UnmatchedMatched	-11.35–5.46	-10.08–6.64	-1.27 1.18	0.85 0.63	11.00 3.50	0.16 0.10
Asset index in 1996 ^*c*^	UnmatchedMatched	-0.63–0.73	-0.60–0.99	0.02 0.27	0.09 0.09	0.29 0.30	0.07 0.07
Simple count of assets in 1996	UnmatchedMatched	3.47 3.29	3.54 2.19	0.06 1.10	0.88 0.09	0.51 0.60	0.07 0.08
D—Resident on parcel in 1996	UnmatchedMatched	0.26 0.25	0.45 0.25	0.19 0.00	0.01 1.00	0.18 0.00	0.09 0.00
Head of cattle on parcel in 1996	UnmatchedMatched	16.41 11.04	31.90 18.52	15.49 7.47	0.03 0.18	19.20 7.72	0.20 0.14
D- Hired workers in 1996	UnmatchedMatched	0.54 0.50	0.36 0.50	0.18 0.00	0.03 1.00	0.18 0.00	0.09 0.00

^*a*^ D indicates a “dummy” variable, coded as 1 = statement true for the respondent, and 0 = statement false for respondent; the mean for these variables is therefore the percentage of respondents for whom statement is true.

^*b*^ Steep slope indicates too steep to plant with crops.

^*c*^ Defined in footnote a of Table 1.

^*d*^ Unmatched sample includes 50 PSA participants and 152 non-participants. Matched sample includes 43 PSA participants and 43 non-participants.

^*e*^ Weighted means for matched controls.

^***f***^ Mean (for categorical covariate) or median (for continuous covariate) difference in the empirical quantile-quantile plot of treatment and control groups on the scale in which the covariate is measured (values > 0 indicate deviations between the groups in some part of the empirical distribution).

^*g*^ Mean eCDF = mean differences in empirical cumulative distribution function (values > 0 indicate deviations between the groups in some part of the empirical distribution).

#### Confounders

A key problem plaguing observational studies is that confounding factors may bias the estimates of treatment effects [46]. Confounding factors affect both program participation and program outcomes. Previous research in Costa Rica has determined that participation in PSA is influenced by factors such as farm size, human capital, and household socio-economic status [13,14,28]. Similar factors are associated with the probability of participation in *Sarapiquí* [27]. Specifically, FUNDECOR gave priority to areas based on watershed protection and recharge, existing protected areas, land use capacity, and biological corridors [39].

We designed the survey instrument to obtain data on these factors that we expected to explain both PSA participation and program outcomes. We obtained input from expert review, reviews of FUNDECOR and FONAFIFO records, pre-tests, and most critically, qualitative case studies [19]. The final survey instrument elicited information about landowner socio-economic characteristics (e.g. age, education, city of origin, current residence, assets, household composition), property (e.g. size, location, soil quality, slope) and land management (e.g. land titling, previous farming experience, participation in previous forest programs, hired and family labor, area under different land uses, livestock, fencing). The survey protocol, including sampling methods, survey instrument, and oral informed consent procedure, was approved by administrative review of the IRB at North Carolina State University (#151-05-6). Survey participants also provided their verbal informed consent to participate in this study.

Interviewers asked about current conditions and conditions in 1996. We selected 1996 because it was the year prior to the launch of the PSA program and memorable to respondents due to its association with Hurricane Cesar and the World Cup in Mexico. While recall data are subject to biases [47], they do provide useful information on prior conditions [42], allowing us to net out unobservable differences across participants and non-participants. We also estimated impact on asset index in 2005, as a robustness check, and found same results regardless of whether we consider 2005 outcome, or change in outcome between 2005 and 1996.

To address incomplete responses, we employed a multiple multivariate imputation process [48–50]. Multiple imputation uses the available information on observations that contain missing values, which can lead to smaller confidence intervals. The imputation process does not depend on imputing the "right" values of the missing variables for individual observations, but rather on correctly modeling their distribution conditional on the observed data. Imputation was done in STATA (v12) using the “switching regression” method of multiple multivariate imputation and averaging across fifty imputed copies of the complete data set. The summary statistics presented in Tables 1 and 2 incorporate these imputed values.

### Empirical Strategy

We assess the impacts of PSA on several indicators of wellbeing and livelihoods using three strategies: (a) compare means, (b) conduct statistical matching, and (c) adjust for bias remaining after matching with multivariate regressions. These progressively eliminate any potential bias from confounders. They are described in detail after a brief summary of how the outcome indicators are constructed.

#### Outcome indicators

We follow established practice e.g. [40,41,51–53] to construct measures of changes in quality of life using asset ownership. Specifically, we use both the count and the first principal component of reported ownership of a car, motorcycle, bicycle, landline telephone, mobile phone, television, microwave, refrigerator and radio. We follow [54] by pooling the data for 1996 and 2005 before applying PCA to generate weights for the asset index. We use the polychoric PCA procedure introduced by [43] for discrete data. The first principal component, which captures the most variance in asset ownership across households, is used to construct the asset index: factor scores from the first component are used as weights for each asset, which are then combined into index scores for 1996 and 2005 cf. [52].

While assets are a commonly used measure of socioeconomic status, they may not be very sensitive to changes in farm income if households choose to adjust current consumption first. In our case, more than a third of PSA participants reported that they used the payments from PSA for consumption. To capture wellbeing changes not reflected in assets, we asked respondents to assess how their quality of life had changed since 1996.

To assess whether PSA payments influenced livelihood strategies, we also examined the proportion of landowners living off-farm before and after the program. PSA could encourage households to move off-farm, by providing a secure source of income during the transition and by limiting production activities on the farm. Alternatively, participants could invest in farm production activities, and in fact, nearly a quarter of participants in our survey said that they used the payments to invest in their farms. We focused on two indicators that respondents could reasonably be expected to recall from nine years prior to the survey: whether they hired workers and the size of their cattle herd.

#### Estimation approaches

First, we compare changes among PSA recipients (treated) with changes among a matched sample of landowners not participating in PSA (controls). The key identification assumption is that mean trend among the controls is equal to the (unobserved) mean trend among the treated in the absence of the PSA program. This is a plausible assumption because we sampled farms eligible for PSA based on geographic rules that ensured they faced similar biophysical and market conditions (a form of “pre-matching”). Nonetheless, there could still be systematic differences in characteristics correlated with both PSA participation and changes over time in livelihoods and welfare.

Second, to eliminate potential confounding, we employ statistical matching procedures, an increasingly common strategy in the impact evaluation literature [55,56]. That is, we identify a matched control group of non-participants that are observationally similar to the treatment group of PSA participants in terms of characteristics that not only drive PSA participation, but also affect program outcomes. The control group provides the basis for estimating livelihood and welfare without the PSA program (the counterfactual), while the treatment group reflects changes in livelihood and welfare due to the policy change. We followed [27] in matching on 1992 forest cover, farm size, participation in past forestry programs, distance to forestry office, percent of farm with steep slopes, whether their forest was fenced, and forest cover change 1986–1992. Further, we match on the baseline (1996) levels of all of the welfare outcomes: asset index, simple sum of assets, residence on or off farm, head of cattle, and whether hired labor to work on the farm.

Third, matching often does not eliminate all differences in the distribution of covariates among the treated and control observations. To reduce any remaining bias, we estimate multivariate regressions using the matched data. Because the covariate distributions are likely to be similar in the matched sample, we do not expect to find statistically significant coefficients on any covariates other than participation in PSA, and we do not use the results to extrapolate out of sample. This strategy of post-matching regression adjustment typically generates treatment effects estimates that are more accurate and more robust to misspecification than parametric regression alone [55,56].

## Results

Despite pre-matching by selecting non-participants in close proximity to participants in the PSA program, we find differences between the two sub-samples in 1996, as shown in the unmatched rows of Table 2. At the 5% significance level, landowners with PSA contracts had more forest (based on interpretation of aerial photographs from 1992), more steeply sloped land, and fewer cattle. In 1996, they were also more likely to have participated in other forest incentive programs, less likely to have fenced their forest, more likely to hire farm workers, and less likely to live on their farms. Turning to changes in welfare between 1996 and 2005, we find no statistically significant differences across participants and non-participants in PSA (Table 1). One possible explanation is that the differences between participants and non-participants (Table 2) mask any welfare gains from participation. Thus, to estimate the causal effect of participation, we need to control for these differences.

**Fig 2:**
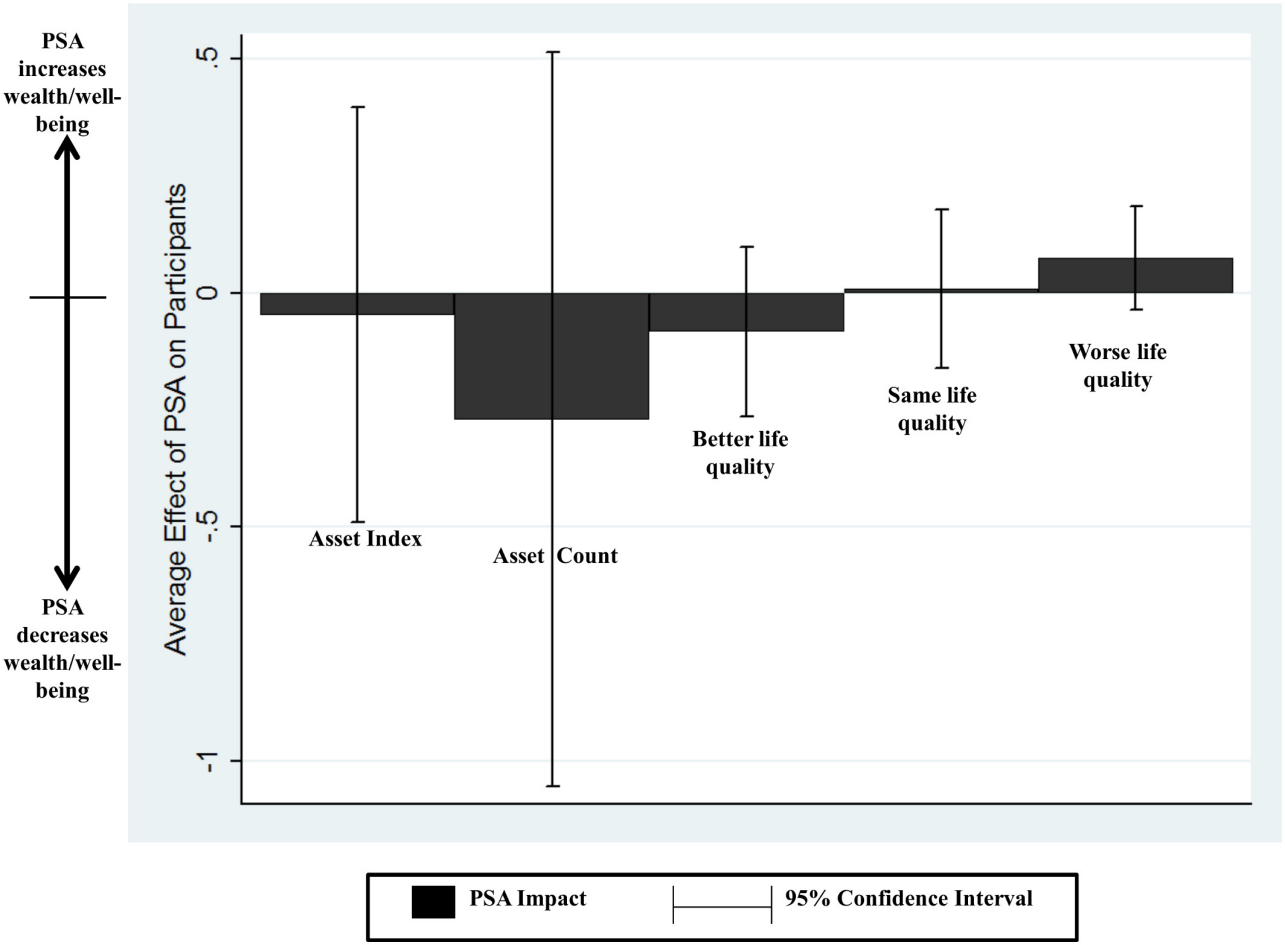
Estimated impacts of PSA between 1996 and 2005 on assets and self-reported well being.

Estimation results from our three ways of measuring the impacts of PSA on welfare are presented in Table 3 and Fig 2. These are the differences in means in the full sample (repeated from Table 1), the differences in means in the matched sample, and the marginal effect estimated in a multivariate regression on the asset index and count (using OLS) and the change in quality of life (using ordered logit) in the matched sample. We consider the post-matching regression results to be the most reliable, because matching greatly reduces differences in the distributions of all covariates except the baseline levels of the asset index and count, as shown by the differences in the means, the raw eQQ, and the mean eCDF in Table 2 [55]. However, differences in the asset index and count are not eliminated by matching, making it important to control for these in post-matching regression. These results are summarized in Fig 1, showing that PSA has no causal impact on any of the welfare measures.

**Table 3:** Estimated impacts of PSA on changes in welfare. Robust standard errors in parenthesis.

	Change in Asset Index(2005 Index—1996 Index)	Change in Asset Count (2005 Count—1996 Count)	Family’s Quality of Life *Better* in 2005 than 1996	Family’s Quality of Life the *Same* in 2005 compared to 1996	Family’s Quality of Life *Worse* in 2005 than 1996
Full sample^a^					
Difference in means^b^	-0.215 (0.174)	-0.369 (0.288)	0.069 (0.075)	-0.129 (0.064)	0.081 (0.049)
Sample selected by covariate matching with calipers^c^					
Difference in means^b^	-0.047 (0.225)	-0.270 (0.397)	-0.083 (0.092)	0.008 (0.086)	0.075 (0.056)
*N* treated dropped by calipers	10	10	10	10	10
Marginal effect from multivariate regression	-0.097 (0.243) ^*d*^	-0.285 (0.398) ^*d*^	-0.095 (0.098) ^*e*^	0.034 (0.084) ^*e*^	0.061 (0.068) ^*e*^

^*a*^ Full sample, with N treated = 50 and N controls = 152.

^*b*^ Statistical significance evaluated with a two-sided *t*-test of the difference in means between treated and control sub-samples.

^*c*^ Calipers restrict matches to units within two standard deviations of each covariate, resulting in matched sample of N treated = 40 treatment and N controls = 40.

^*d*^ Ordinary least squares regression on change in consumer durables, with all variables used in matching as covariates.

^*e*^ Ordered logit regression on change in quality of life, with all variables used in matching as covariates.

**Fig 3:**
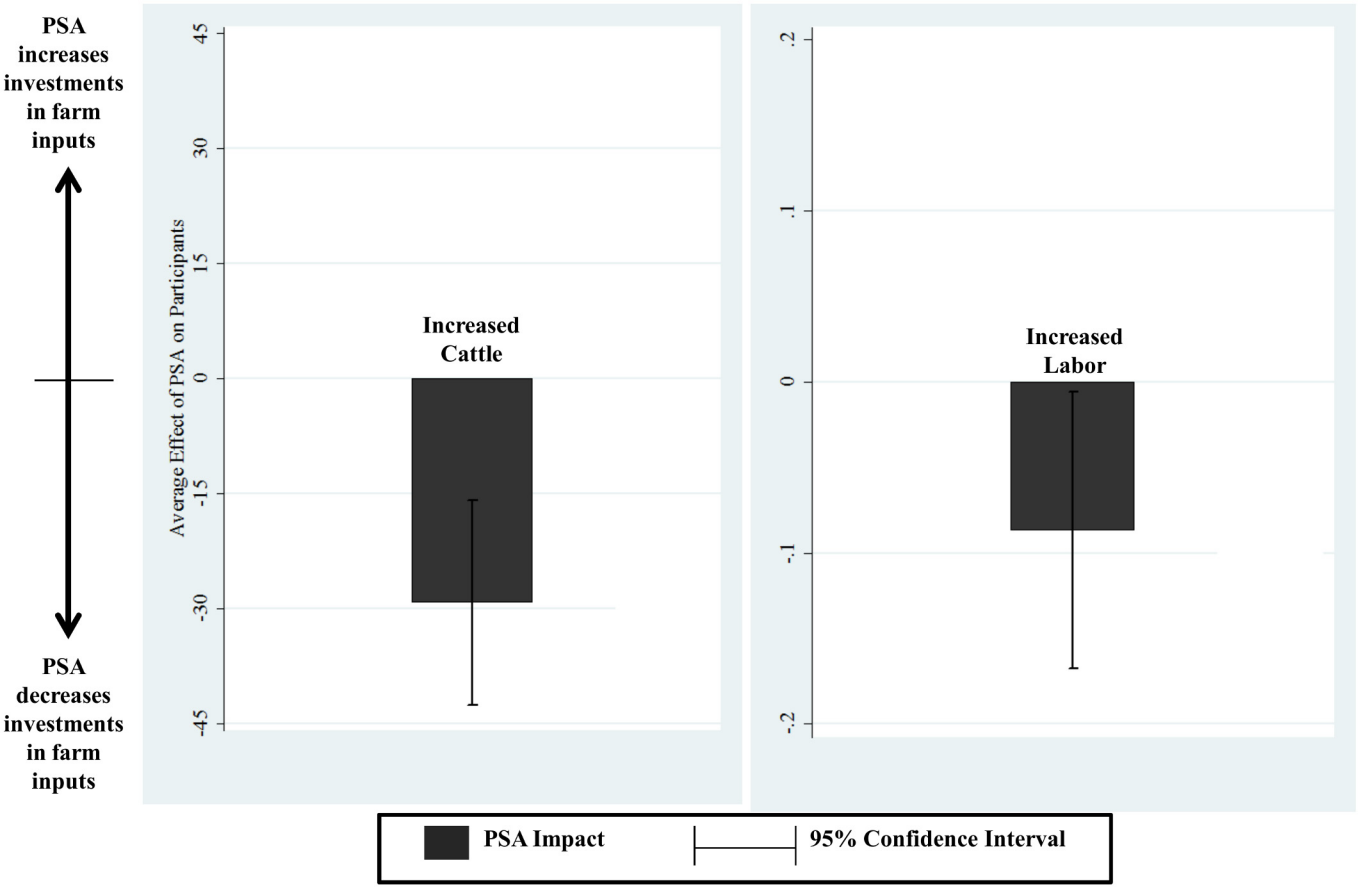
Estimated impacts of PSA between 1996 and 2005 on changes in farm investment.

This raises the question of whether landowners instead use the cash payments to shift livelihoods in ways that either support or undermine the forest conservation goals of PSA. Therefore, we estimate the impacts of PSA on the probability that the landowner moves off the farm, the probability that the landowner starts to hire farm labor, and the number of cattle on the farm. The results from post-matching regression are presented in Table 4 and Fig 3. Participation in PSA did not affect the probability of moving off-farm and reduced both the number of cattle and the probability of hiring farm labor. In particular, the marginal effect on cattle is large (relative to average herd sizes) and important because of the role of cattle in deforestation and forest degradation [57]. While this is consistent with the positive impact on forest cover found by [27], it does not suggest an increase in welfare and therefore still begs the question of why landowners voluntarily participate in the program.

**Table 4:** Estimated impacts of PSA on changes in residence and farm investments. Covariates in all regressions are all variables used in matching, including baseline 1996 measures of outcomes.

	Post-matching with calipers^c^		
	Marginal effect	St Error	P-value
*Changes in cattle owned* ^*a*^			
Cattle in 2005 –cattle in 1996	-29.284	6.754	0.000
*Changes in hired labor* ^*b*^			
Hired labor in 1996 → No hired labor in 2005	0.043	0.026	0.097
Hired labor in 1996 → Hired labor in 2005	-0.186	0.077	0.015
No hired labor in 1996 → No hired labor in 2005	0.229	0.089	0.010
No hired labor in 1996 → Hired labor in 2005	-0.087	0.041	0.036
*Changes in residence* ^*b*^			
Off-farm in 1996 → On-farm in 2005	-0.040	0.034	0.236
Off-farm in 1996 → Off-farm in 2005	-0.060	0.052	0.246
On-farm in 1996 → On-farm in 2005	0.086	0.069	0.215
On-farm in 1996 → Off-farm in 2005	0.014	0.014	0.318

^*a*^ Estimation results from multivariate OLS regression.

^*b*^ Estimation results from multivariate ordered logit regression.

^*c*^ Matching with calipers results in matched sample of 40 treated and 40 control observations.

## Discussion and Conclusions

Despite the PSA program reducing two key on-farm inputs (cattle and hired labor) and transferring a large amount of cash to each household, we do not detect any improvements in household assets or the probability of self-reported improvement in quality of life. There are two caveats on these findings. First, our sample design may not have sufficient statistical power to detect small changes in household wellbeing, especially those measured with binary indicators, although matching is believed to increase the minimum detectable effect size. Second, while our measure of forest cover (including mature and secondary forest) can change within just a few years over large portions of the enrolled farms [27], socio-economic outcomes such as wealth and wellbeing are more likely to change slowly and incrementally. Setting aside these caveats, our results are consistent with the common claim that voluntary PES can induce landowners to supply environmental benefits while doing no harm to their households. However, the Costa Rican PES also does not appear to have benefited participants in any of the dimensions of wellbeing measured in our survey.

Therefore, we must consider why farmers would participate in a voluntary program that does not offer any welfare benefits. Porras et al. [26] show that the costs of enrolling and complying with the program can be significant, and thus one possible explanation is that landowners underestimated those costs. However, we reject this explanation because our sample of participants all re-enrolled in the program after their first five-year contracts expired and thus are likely to have understood the costs. Furthermore, in our study region, FUNDECOR assisted landowners with enrollment, preparation and execution of forest management plans in exchange for a small, known and fixed proportion of the payments. Moreover, most participants (70%) reported in the household survey that they were satisfied with the program and would recommend it to their neighbors. In fact, the program was experiencing excess demand from landowners during our study period.

The more plausible explanation is that the wealth and self-report wellbeing indicators do not measure the full suite of program benefits. For example, [31] found that participation in the program enables households to move off-farm. We do not find any evidence of this in our study region. Thus, we return to some key findings in [19], who examined program participation using a mixed methods approach that combined in-depth case studies, household surveys, expert interviews, and literature reviews. In that study, participants reported joining the program to secure their property rights and to contribute to forest conservation. Interviews with forestry officials yielded similar claims: landowners with higher levels of environmental consciousness tended to enroll in the program and owners of large properties enrolled in PSA “to protect” their land from aggressive “land development” policies by the Costa Rican Institute of Agricultural Development (*Instituto de Desarrollo Agrario*, IDA). Specifically, key informants reported that during our study period, IDA officials believed that forests were “useless lands” and so should be available for seizure and agricultural development by farmers. Our survey also elicited motivations for participation in PSA. More than 50% of participants in the sample mentioned environmental protection as an important reason for enrolling in the program.

In sum, we respond to recent calls for more careful evaluation of conservation policies e.g. [58,59] making the following contributions to a slow-growing empirical literature on the impacts of conservation programs. First, in contrast to impact evaluations focused on deforestation, we focus on social outcomes that are also likely to explain program participation and conservation outcomes. Although PES is often viewed primarily as an environmental program, the potential for high spatial overlap of biological diversity and poor vulnerable populations across the tropics [60,61] has led to PES being widely discussed as a mechanism for promoting rural development [62,63]. However, there are relatively few empirical studies of the socio-economic impacts of PES. Second, unlike most evaluations relying on secondary and remotely sensed data, which could be subject to problems of ecological fallacy if used to describe farm households, we take a microeconomics perspective by conducting and analyzing data from a primary household survey. Third, the analyses and interpretations reported in this paper draw on and complement the findings from companion papers. For example, our estimates of reductions in farm investments reveal potential mechanisms through which PES could induce greater forest cover, thus providing more confidence in a previous claim that PES increased forest cover on participating farms [27]. Similarly, we find that explaining the continued participation by farmers in PES (despite no detectable improvements in assets and wellbeing) requires looking beyond simple economic rationales and material outcomes. Combined with previous qualitative case studies, our survey results suggest that farmers participated in order to gain recognition of their conservation efforts, which both made them feel more secure about their land tenure and gave them a “warm glow” from conserving forests [19].

Thus, we find that the Costa Rican PES program “does no harm” in terms of traditional measures of welfare, such as asset indices, and may deliver less tangible benefits that require researchers to look beyond simple economic rationales and material outcomes in order to understand the social impacts of nature conservation.

## Supporting Information

S1 File Data. This file contains all data necessary to replicate the underlying findings reported in this paper.
(TXT)
