# Correction: Do Payments Pay Off? Evidence from Participation in Costa Rica's PES Program

**DOI:** 10.1371/journal.pone.0136809

**Published:** 2015-08-24

**Authors:** R. A. Arriagada, E. O. Sills, P. J. Ferraro, S. K. Pattanayak

In Table 2, the matched and unmatched samples and their values have been combined. Please see the correct Table 2 here.

**Table 2:** Covariate Balance.

Variable ^a^	Sample ^d^	Mean Value PSA	Mean Value Non-PSA ^e^	Diff Mean Value	p-value	Raw eQQ Diff ^f^	Mean eCDF Diff ^*g*^
Total native forest in 1992 (ha)	Unmatched	86.13	37.14	48.99	0.02	45.82	0.14
	Matched	51.73	45.89	5.84	0.50	8.79	0.05
Farm size (ha)	Unmatched	165.11	71.43	93.68	0.06	90.05	0.19
	Matched	81.08	73.78	7.31	0.64	18.39	0.10
D—Previous participation in other forest programs	Unmatched	0.32	0.25	0.07	0.01	0.18	0.09
	Matched	0.25	0.25	0.00	1.00	0.00	0.00
Distance to forestry office (km)	Unmatched	29.48	25.08	4.41	0.05	4.68	0.08
	Matched	30.64	28.21	2.43	0.19	3.82	0.05
Percent farm on steep slope ^b^	Unmatched	38.40	25.54	12.86	0.01	13.01	0.13
	Matched	37.25	34.01	3.24	0.28	4.49	0.05
D—Forest fenced in 1996	Unmatched	0.20	0.50	-0.30	0.00	0.30	0.15
	Matched	0.15	0.15	0.00	1.00	0.00	0.00
Hectares of forest in 1992 minus hectares of forest in 1986	Unmatched	-11.35	-10.08	-1.27	0.85	11.00	0.16
	Matched	-5.46	-6.64	1.18	0.63	3.50	0.10
Asset index in 1996 ^c^	Unmatched	-0.63	-0.60	0.02	0.09	0.29	0.07
	Matched	-0.73	-0.99	0.27	0.09	0.30	0.07
Simple count of assets in 1996	Unmatched	3.47	3.54	0.06	0.88	0.51	0.07
	Matched	3.29	2.19	1.10	0.09	0.60	0.08
D—Resident on parcel in 1996	Unmatched	0.26	0.45	0.19	0.01	0.18	0.09
	Matched	0.25	0.25	0.00	1.00	0.00	0.00
Head of cattle on parcel in 1996	Unmatched	16.41	31.90	15.49	0.03	19.20	0.20
	Matched	11.04	18.52	7.47	0.18	7.72	0.14
D- Hired workers in 1996	Unmatched	0.54	0.36	0.18	0.03	0.18	0.09
	Matched	0.50	0.50	0.00	1.00	0.00	0.00

^*a*^ D indicates a “dummy” variable, coded as 1 = statement true for the respondent, and 0 = statement false for respondent; the mean for these variables is therefore the percentage of respondents for whom statement is true.

^*b*^ Steep slope indicates too steep to plant with crops.

^*c*^ Defined in footnote a of Table 1.

^*d*^ Unmatched sample includes 50 PSA participants and 152 non-participants. Matched sample includes 43 PSA participants and 43 non-participants.

^*e*^ Weighted means for matched controls.

^***f***^ Mean (for categorical covariate) or median (for continuous covariate) difference in the empirical quantile-quantile plot of treatment and control groups on the scale in which the covariate is measured (values > 0 indicate deviations between the groups in some part of the empirical distribution).

Note: The seventh and eighth columns present three measures of the differences in the covariate distributions between PSA and non-PSA farms. If matching is effective, all of these measures should move dramatically toward zero (Ho et al., 2007).

## References

[1] Arriagada RA, Sills EO, Ferraro PJ, Pattanayak SK (2015) Do Payments Pay Off? Evidence from Participation in Costa Rica’s PES Program. PLoS ONE 10(7): e0131544 doi: 10.1371/journal.pone.0131544 26162000 10.1371/journal.pone.0131544 PMC4498908

